# Compressing Spin-Polarized ^3^He With a Modified Diaphragm Pump

**DOI:** 10.6028/jres.106.033

**Published:** 2001-08-01

**Authors:** T. R. Gentile, D. R. Rich, A. K. Thompson, W. M. Snow, G. L. Jones

**Affiliations:** National Institute of Standards and Technology, Gaithersburg, MD 20899-8461; Indiana University, Bloomington, IN 47408; Hamilton College, Clinton, NY 13323

**Keywords:** ^3^He, helium, metastability-exchange, MRI, neutron, optical pumping, polarization, spin filter

## Abstract

Nuclear spin-polarized ^3^He gas at pressures on the order of 100 kPa (1 bar) are required for several applications, such as neutron spin filters and magnetic resonance imaging. The metastability-exchange optical pumping (MEOP) method for polarizing ^3^He gas can rapidly produce highly polarized gas, but the best results are obtained at much lower pressure (~0.1 kPa). We describe a compact compression apparatus for polarized gas that is based on a modified commercial diaphragm pump. The gas is polarized by MEOP at a typical pressure of 0.25 kPa (2.5 mbar), and compressed into a storage cell at a typical pressure of 100 kPa. In the storage cell, we have obtained 20 % to 35 % ^3^He polarization using pure ^3^He gas and 35 % to 50 % ^3^He polarization using ^3^He-^4^He mixtures. By maintaining the storage cell at liquid nitrogen temperature during compression, the density has been increased by a factor of four.

## 1. Introduction

Several applications of nuclear spin-polarized ^3^He gas such as neutron polarizers [1–5], polarized gas magnetic resonance imaging (MRI) [6,7], and polarized targets [8] require gas pressures on the order of 100 kPa (1 bar). Two optical pumping methods have been employed to produce polarized gas for these applications: spin-exchange optical pumping (SEOP), [9–12] in which the gas is polarized directly at high pressure, and metastability-exchange optical pumping (MEOP) [13–15], in which the gas is polarized at low pressure (~0.1 kPa) and then compressed. Compression of polarized ^3^He, produced by MEOP using a helium lamp, was first demonstrated thirty years ago using a Toepler pump [16]. Following the development of the arc lamp pumped Nd:LMA laser [17,18], further development of this approach was pursued at the University of Mainz in the early 1990’s [19]. Soon therafter, the Mainz group developed a two-stage piston compressor for polarized ^3^He [20], which has subsequently been applied to electron scattering experiments, neutron polarizers and polarized gas MRI [21]. This apparatus can compress po-larized ^3^He gas to pressures of several hundred kPa at a rate of 50 kPa·L/h while maintaining a polarization of 55 % [2]. These impressive results have motivated us to develop an apparatus of similar design [3]. However, these apparatus are large and complex, so in parallel we are pursuing a compact and simple apparatus for polarized gas compression, which is the subject of this paper. The compact compression apparatus is based on modification of a commercial diaphragm pump. Some description of this apparatus and the first applications to neutron spin filters and polarized gas MRI have recently been reported [1,22,23]. In this paper, we present a detailed description of the apparatus, techniques to optimize its performance and increase its capability, and updated results. We expect that it is only the first step in the direction of compact compression methods.

The two optical pumping methods, spin-exchange and metastability-exchange, each have their respective attributes and difficulties. We are pursuing both methods for the emerging fields of ^3^He-based neutron spin filters and polarized gas MRI. The attributes of spin-exchange include simplicity, small size, and compatibility with continuous, long term operation, while the difficulties include an inherently slow polarized ^3^He production rate and optical pumping-related constraints on the cell pressure. The physics of the metastability-exchange method allows for rapid production of highly polarized gas at low pressure, but the user is burdened with the task of preserving the polarization during compression. The present compression devices are large and complex, which has limited the number of groups employing this method and effectively prohibited the installation of such devices directly onto neutron beam lines or into other *in situ* arrangements. The continuing motivation for the work begun in this paper is to develop a compact, simple, and reliable compression apparatus.

In the metastability-exchange method, electronic polarization is produced by optical pumping of metastable helium atoms, and the polarization is rapidly transferred to the nucleus of the metastable atom via the hyperfine interaction. The electronic excitation in the metastable atom is transferred to a ground state atom during a collision, while the nuclear polarization is unperturbed. Hence the collision results in a nuclear spin-polarized ground state atom, and the newly excited metastable atom is then repolarized by laser light.

**Fig. 1:**
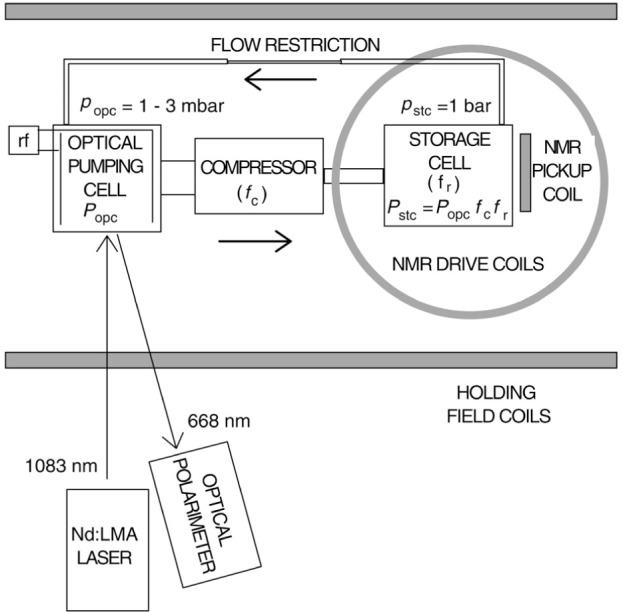
Conceptual diagram of the apparatus. The notation is discussed in Sec. 2.

As shown in Fig. 1 the apparatus can be divided into three stages: optical pumping of low pressure gas (0.1 kPa to 0.3 kPa), compression, and storage of the high pressure gas (100 kPa). The apparatus is immersed in a uniform magnetic field produced by two 82 cm ID holding field coils in the Helmholtz configuration. Metastable atoms are produced by a weak electrodeless radio-frequency (rf) discharge, and optically pumped by light at a wavelength of 1083 nm. The gas can either be accumulated in the storage cell (“fill mode”), or a constant pressure can be maintained in the storage cell by continuously leaking gas back to the optical pumping cell (“recirculation mode”). In the optical pumping cell, the polarization is determined from analysis of the circular polarization of 668 nm wavelength light emitted from the discharge. In the storage cell, NMR (nuclear magnetic resonance) provides a signal that is proportional to the magnetization. An absolute measure of the storage cell polarization is obtained by optically pumping gas at low pressure in the storage cell and calibrating the NMR system against optical polarimetry.

The paper is organized as follows: In Sec. 2 we present the basic principles that influence the achievable polarization of the compressed gas. In Sec. 3 we present two schemes for optimizing the optical pumping efficiency in a compact system. The details of the apparatus are described in Sec. 4 and the results in Sec. 5. In Sec. 6 we summarize the status of this work and discuss the outlook for future development.

## 2. Principles

In this section we discuss the basic principles that influence the achievable polarization of the compressed gas. In the analysis that follows, we consider the steady-state case of continuous recirculation of the polarized gas. The achievable gas polarization in the storage cell (StC), *P*_stc_, is given by

$$P_{\text{stc}}=P_{\text{opc}}f_{c}f_{r}$$ (1)

where *P*_opc_ is the gas polarization in the optical pumping cell (OPC), *f*_c_ is the fraction of *P*_opc_ preserved in transit from the OPC to the StC, and *f*_r_ is the fraction of *P*_stc_ maintained given the finite relaxation time in the StC. *f*_r_ is given by

$$f_{r}=\frac{1}{1+(t_{\text{stc}}/\tau_{\text{stc}})}$$ (2)

where *t*_stc_ is the mean residence time in the storage cell and stc is the relaxation time of the polarization in the storage cell. *f*_r_ is usually close to one and therefore is not a major consideration in the achievable value of *P*_stc_. As discussed further in Sec. 5.1, *f*_c_ decreases with increasing time in the compressor. Typically we operate at a sufficiently high throughput to obtain *f*_c_ ≈ 0.75.

For a single optical pumping cell, *P*_opc_ is given by

$$P_{\text{opc}}=\frac{P_{0}}{1+(\tau /t_{\text{opc}})}$$ (3)

where *P*_0_ is the achievable optical pumping cell polarization for zero throughput, **τ** is the effective optical pumping time constant [14] and *t*_opc_ is the mean residence time in the optical pumping cell. (In the interest of simplicity we have not included the known dependence of **τ** on *P*_opc_ [14,24], but we will revisit this issue in the discussion of results in Sec. 5.) In principle *P*_opc_ could be optimized by simply increasing the time in the optical pumping cell (*t*_opc_), but the increased polarization loss in the compressor (*f*_c_) with decreased throughput precludes this simple solution. Optimal operation is a balance between maximizing *P*_opc_ (low flow) and maximizing *f*_c_ (high flow), hence it is desirable to maintain high values of *P*_opc_ at relatively high throughput. For the most part, the inherently rapid optical pumping rate of the metastable method with current laser technology [14] fulfills this requirement. We have developed two schemes to maximize the efficiency of the optical pumping while preserving the compact spirit of the apparatus: 1) series optical pumping cells with a diffusion restriction, and 2) polarization preserving recirculation (PPR). In scheme (1), the gas passes sequentially through two optical pumping cells, the first OPC serving as a “pre-polarizer” for the second OPC. The two cells are separated by a diffusion restriction, which yields a higher value for the polarization in the second optical pumping cell, *P*_opc2_, than would be obtained with free exchange between the two cells. In scheme (2), the polarization of the gas is preserved during recirculation from the StC back to the OPC. These schemes are discussed in detail in Sec. 3, but we note that they are not critical to the basic operation of this apparatus.

For pure ^3^He gas, the most important factor that influences the value of *P*_0_ in this apparatus is the OPC pressure. The optimum pressure for optical pumping of pure ^3^He is about 0.05 kPa [14], whereas in this apparatus we obtain a typical OPC pressure of 0.25 kPa. In this range of pressure, we obtain higher values of the ^3^He polarization by optically pumping mixtures of ^3^He and ^4^He [13]. For such mixtures (which are suitable for neutron spin filters because ^4^He is transparent to neutrons), the relatively high OPC pressure is less significant an issue than for pure ^3^He.

Because of the limited time in the OPC, the optimum discharge intensity and frequency is determined by a balance between maximizing *P*_0_ and minimizing **τ**. At our typical OPC pressure, the highest value of *P*_0_ would be obtained with a weak, low frequency (0.1 MHz to 1 MHz) discharge, whereas the smallest value of **τ** would be obtained with a strong, high frequency (~10 MHz) discharge [13,14]. For ^3^He-^4^He gas mixtures with ^3^He concentrations of 25 % to 50 %, we have obtained values of *P*_0_ between 0.8 and 0.6 for sealed cells filled to a total pressure of 0.21 kPa. The achievable value of *P*_0_ depends primarily on the discharge intensity, but also on the discharge frequency and ^3^He concentration. Because minimizing is important in this apparatus, we use a high discharge frequency (13.6 MHz) and a fairly strong discharge. For operation of the apparatus with ^3^He-^4^He gas mixtures, typical values of *P*_0_, *P*_opc_, and *P*_stc_ are 0.55 to 0.65, 0.45 to 0.65, and 0.30 to 0.50, respectively. For pure ^3^He gas, the corresponding typical values are 0.40 to 0.50, 0.25 to 0.45, and 0.20 to 0.35, respectively. More detailed information is provided in Sec. 5.

Here we summarize the notation used in this paper:

- *P*_opc_ = polarization in a single optical pumping cell
- *f*_c_ = fraction of polarization preserved in transit from the OPC to the StC
- *f*_r_ = fraction of *P*_stc_ maintained given the finite relaxation time in the StC
- *P*_stc_ = polarization in the storage cell
- *P*_0_ = polarization in a single OPC for zero throughput
- **τ** = effective optical pumping time constant in a single optical pumping cell
- **τ**_1_ (**τ**_2_) = effective optical pumping time constant in OPC1 (OPC2)
- *t*_opc_ (*t*_stc_) = residence time in a single optical pumping cell (storage cell)
- *t*_opc1_ (*t*_opc2_) = residence times in OPC1, OPC2
- **τ**_stc_ = relaxation time in the storage cell
- *P*_opc1_ (*P*_opc2_) = polarization in OPC1 (OPC2)
- *f P*_0_ (*P*_0_) = polarization in OPC1 (OPC2) for zero throughput
- *p*_opc1_ (*p*_opc2_) = pressure in OPC1 (OPC2)
- *p*_stc_ = pressure in the storage cell
- *Q* = throughput (kPa·L/s)
- *F* = volume flow rate at the outlet of the second stage of the compressor (cm^3^/s)

## 3. Optical Pumping Schemes

### 3.1 Series Optical Pumping Cells with Diffusion Restriction

**Fig. 2:**
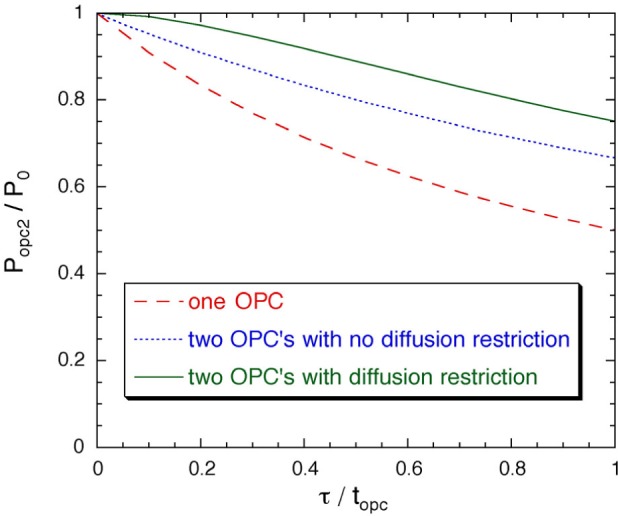
The variation of *P*_opc2_ with the ratio τ/*t*_opc_, for three cases: 1) determined from Eq. (4) for *f* = 1, τ= τ_1_ = τ_2_ and *t*_opc_ = *t*_opc1_ = *t*_opc2_, which corresponds to a two-cell arrangement with a diffusion restriction (solid line); 2) *P*_opc_ determined from Eq. (3), which corresponds to a single cell (dashed line); and 3) *P*_opc_ determined from Eq. (3) with unchanged and *t*_opc_ doubled, which corresponds to the best possible case of a two-cell arrangement with no diffusion restriction (dotted line).

Because the ^3^He gas is optically thin, the simplest approach to increasing the optical pumping efficiency is to make the cell longer, which increases the residence time in the OPC without a proportional increase in **τ**. (For larger cell diameter the increases in residence time and **τ** have been observed to be comparable, yielding a minimal increase in efficiency [14].) However, employing a longer cell also increases the length of the uniform magnetic field required to prevent relaxation due to field gradients [25]. Given the small size of the diaphragm compressor, we preferred a scheme that would keep the entire apparatus compact. One option is use two adjacent optical pumping cells that are connected by a tube, which yields a doubling of the optical path length while the physical length of each cell is unchanged. The overall efficiency of such a two-cell arrangement is improved if one adds a diffusion restriction between the two cells because the gas entering the second cell has been pre-polarized in the first cell. An equilibrium polarization is established in the first optical pumping cell, and a new equilibrium is established in the second cell. The polarization in the second cell is given by

$$P_{\text{opc}2}=P_{\text{opc}1}+\frac{P_{0}-P_{\text{opc}1}}{1+(\tau_{2}/t_{\text{opc}2})}=P_{0}\frac{1+(\tau_{1}/t_{\text{opc}1})+(f\tau_{2}/t_{\text{opc}2})}{\left[1+(\tau_{1}/t_{\text{opc}1})][1+(\tau_{2}/t_{\text{opc}2})\right]}$$ (4)

where *P*_0_ (*f P*_0_) is the achievable optical pumping cell polarization for zero throughput in OPC2 (OPC1), ***τ***_2_ (***τ***_1_) is the effective optical pumping time constant in OPC2 (OPC1), and *t*_opc2_ (*t*_opc1_) is the residence time in OPC2 (OPC1). We have used different parameters for each cell because the pressure, laser power, and discharge intensity obtained in OPC1 may differ from that obtained in OPC2. *P*_opc1_ is given by Eq. (3) for the parameters relevant to OPC1. For illustration, we assume that the parameters are the same in each cell, ie., *f* = 1, ***τ*** = **τ**_1_ = **τ**_2_ and *t*_opc_ = *t*_opc1_ = *t*_opc2_. Fig. 2 shows the variation of *P*_opc2_ with the ratio **τ**/*t*_opc_, as determined from Eq. (4), and compares it to two other cases: 1) *P*_opc_ determined from Eq. (3), which corresponds to a single cell, and 2) *P*_opc_ determined from Eq. (3) with unchanged and *t*_opc_ doubled, which corresponds to the best possible case of a two-cell arrangement with no diffusion restriction. The two-cell arrangement with a diffusion restriction yields the best results.

Realizing such a diffusion restriction without increasing *p*_opc1_ excessively is possible because the diffusion rate increases with the square of the connecting tube diameter while the viscous flow conductance increases as the fourth power of the diameter. The diffusion time between two cells of volume *V* connected by a capillary of diameter *d* and length *L* (in cm) is given by *T*_d_ = 4*LV*/π*d*^2^*D* [26], where *D* = 190/*p* is the diffusion coefficient in cm^3^/s for ^3^He at a pressure *p* in kPa [27–30]. The pressure drop between the two cells is given by Δ*p* = *Q*/*C*, where *Q* is the throughput and *C* = 1400*p*_av_*d*^4^/*L* is the viscous flow conductance in L/s for the average pressure *p*_av_ in kPa, and the tube diameter and length in cm [31]. Typical values in the apparatus are *V* = 440 cm^3^, *d* = 0.30 cm, *L* ≈ 40 cm, and *p*_av_ = 0.36 kPa at a throughput of *Q* = 0.015 kPa·L/s, which yields *T*_d_ = 470 s, Δ*p* = 0.15 kPa, and *t*_opc_ = 10 s. (In practice, we observeΔ*p* = 0.2 kPa.) Hence the average residence time in the cell is nearly 50 times shorter than the diffusion time. The resulting value of 0.46 kPa for *p*_opc1_ has been found to be acceptable for optical pumping of mixtures and could be decreased for optical pumping of pure ^3^He. The diffusion restriction does not affect the pressure in OPC2.

### 3.2 Polarization Preserving Recirculation

**Fig. 3:**
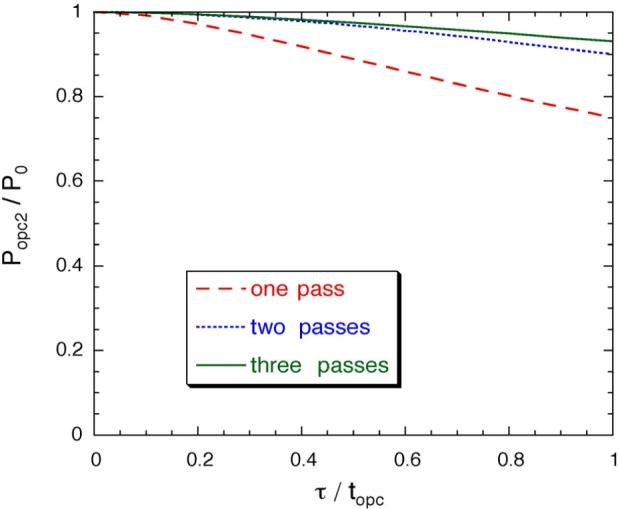
The variation of *P*_opc_ with **τ**/*t*_opc_ for one (dashed line), two (dotted line), and three (solid line) passes through a two-cell arrangement with a diffusion restriction, assuming polarization preserving recirculation (see Sec. 3.2) and *f*_c_ = 0.8.

For recirculation mode, preserving the polarization in its return from the storage cell to the optical pumping cells allows the achievable OPC polarization to potentially increase in each recirculation cycle. For a single optical pumping cell, the value of *P*_opc_ after polarization preserving recirculation (PPR) and a second pass of optical pumping can be determined using Eq. (4) by replacing *P*_opc1_ with *P*_stc_. The calculation can be iterated for more passes through the OPC, and extended to include a two-cell arrangement. The variation of *P*_opc2_ with **τ**/*t*_opc_ is shown in Fig. 3 for one, two, and three passes through a two-cell arrangement with a diffusion restriction, where we have assumed that *f*_c_ = 0.8. It can be seen that two passes are sufficient to obtain most of the possible improvement.

## 4. Apparatus

**Fig. 4:**
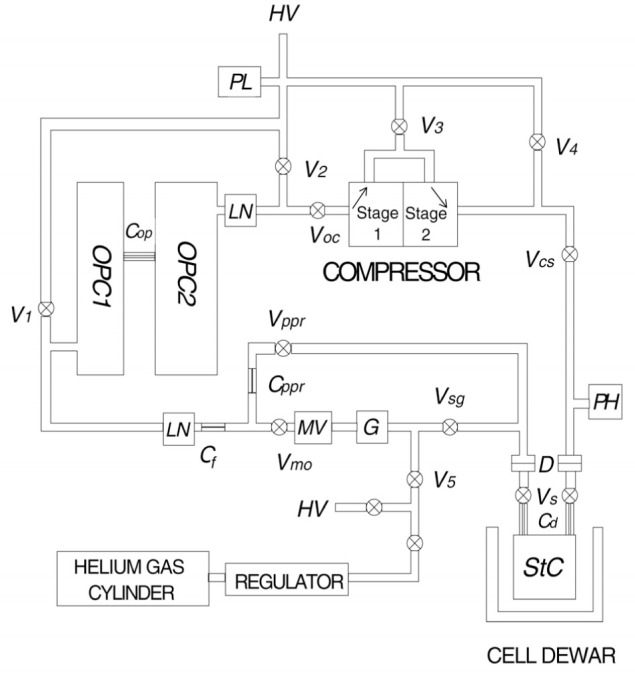
Schematic diagram of the compression apparatus, including the ^3^He (or ^3^He-^4^He mixture) gas cylinder, pressure regulator, glass stopcock valves (*V*_oc_, *V*_cs_, *V*_s_, *V*_sg_, *V*_ppr_, *V*_mo_, *V*_1_-*V*_5_), getter (G), metering valve (MV), flow restricting capillary (*C*_f_), PPR flow-restricting capillary (*C*_ppr_), optical pumping cells (OPC1 and OPC2), diffusion restriction between the OPCs (*C*_op_), liquid nitrogen traps (LN), the modified two-stage diaphragm compressor, a capacitance manometer to measure the optical pumping cell pressure (PL), a strain gauge sensor to measure the storage cell pressure (PH), the storage cell (StC), glass O-ring connections to permit detachment of the StC (D), storage cell diffusion-restricting capillaries (*C*_d_), and the cell dewar (optional). The system is evacuated by connections to a turbomolecular pump (HV).

**Fig. 5:**
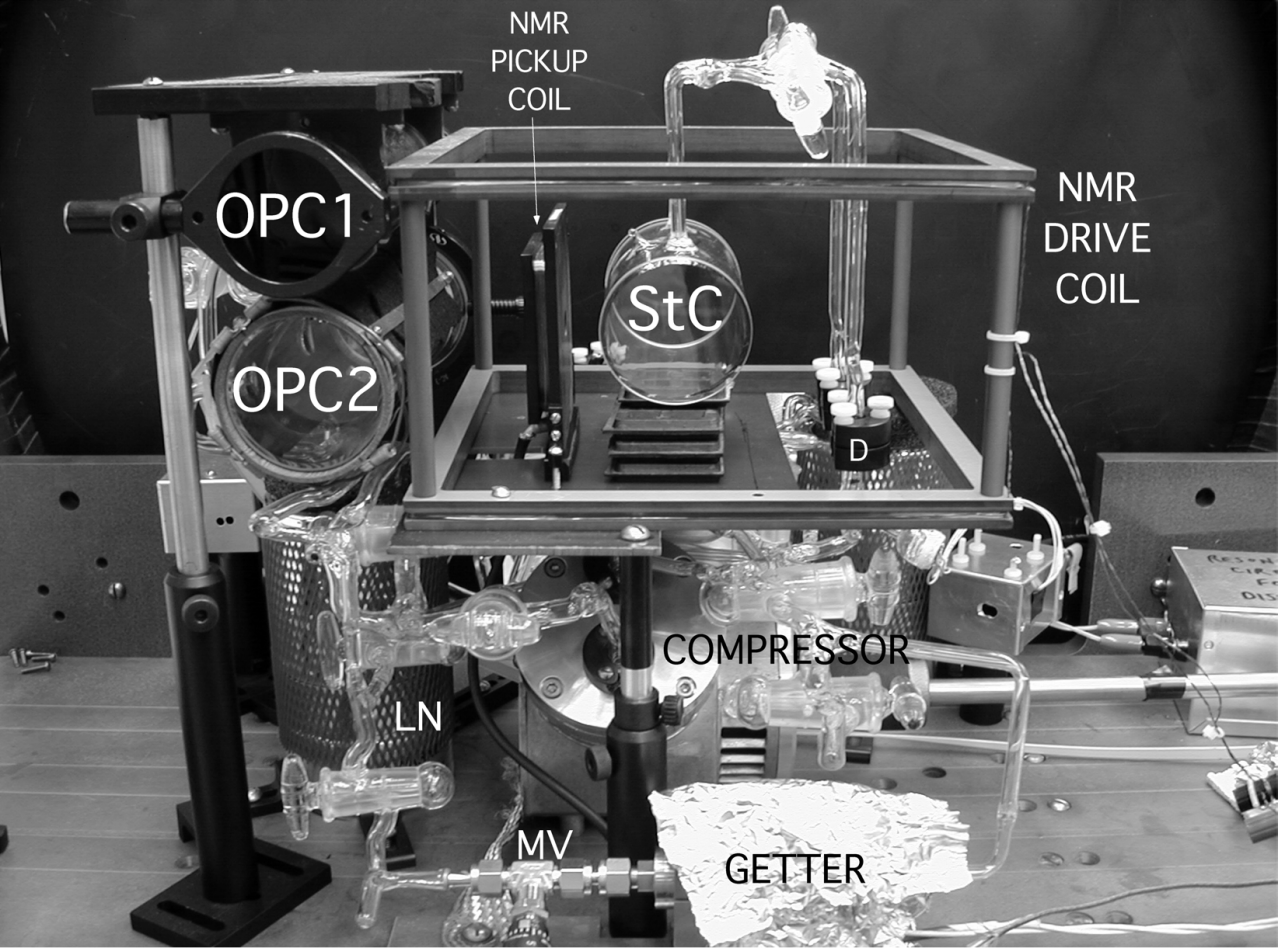
Photograph of the section of the apparatus (about 35 cm on a side) that is immersed in the holding magnetic field. The storage cell is located above the compressor, near the centers of the holding field coils and NMR drive coils. The two OPCs are on the left, and the small dewar for the liquid nitrogen traps are just below the OPCs. The getter (covered with aluminum foil) and metering valve are in the foreground.

A detailed diagram of the compression apparatus is shown in Fig. 4. The gas is polarized in two 20 cm long optical pumping cells (OPC1, 4.7 cm ID and OPC2, 5.9 cm ID) connected by a diffusion-restricting capillary *C*_op_ (see Sec. 3.1), passes through the two-stage diaphragm compressor, and is delivered to a storage cell (StC). The part of the apparatus maintained in a uniform magnetic field, shown in the photograph in Fig. 5, fits into a cube about 35 cm on a side. The storage cell is located at the center of the holding field coils. The cell dewar (not shown in photograph) is simply a styrofoam container, allowing the cell to be maintained at liquid nitrogen temperature during compression. To allow space for the storage cell dewar and the NMR pickup coil, OPC2 is located 13 cm to the side of the axis of the holding field coils, and OPC1 is parallel to OPC2 and immediately above it. Gas purity is maintained by a getter (G) and liquid nitrogen traps (LN). The throughput is controlled by a gas regulator, glass capillaries (*C*_f_, *C*_ppr_), and a metering valve (MV). We discuss the various aspects of the apparatus in detail.

### 4.1 Gas Handling

Connections within the gas recirculation loop are constructed almost entirely of Pyrex glass. 6 mm ID tubing is used to obtain high conductance in the low pressure section and 2 mm ID tubing is used to minimize volume in the high pressure section. Gas flow is controlled using glass stopcock valves, lubricated with a low vapor pressure, bakeable grease [32].^1^ (However, we have yet to bake the recirculation loop.) Glass flanges with Viton O-rings are used to connect glass tubing to the aluminum compressor head. The entire loop is evacuated by connections to a turbomolecular pump (HV), and gas is introduced from a storage bottle through the regulator. Since we typically admit gas to the recirculation loop at subatmospheric pressure, a high purity absolute pressure regulator with a delivery pressure range of 0 kPa to 200 kPa is used. The storage bottles contain either pure ^3^He or premixed ^3^He-^4^He gas mixtures. The connections from the glass recirculation loop to the stainless steel high vacuum system are made with standard glass to metal bellows, outside the holding field coils.

The pressures in the optical pumping cells, *p*_opc1_ and *p*_opc2_, are measured using a capacitance manometer (PL). *p*_opc1_ (*p*_opc2_) is measured with V_1_ (V_2_) open and V_2_ (V_1_) closed; both valves are closed during optical pumping because the capacitance manometer is magnetic and located outside the holding field coils. The StC pressure *p*_stc_ is measured by a non-magnetic, ceramic strain gauge [33] (PH) that was bonded to glass using high vacuum epoxy.

Before operating the apparatus, all valves are opened and the entire system is evacuated with the compressor running. The typical base pressure measured by a gauge near the turbomolecular pump is 3×10^‒5^ Pa. For operation in fill mode, valves V_1_, V_2_, V_3_, V_4_, V_sg_, and V_ppr_ are then closed and ^3^He gas is admitted through valve V_5_. When the desired pressure in the StC is obtained, valve V_cs_ is closed and the compressor is turned off. In recirculation mode, valve V_sg_ is opened and V_5_ is closed. To employ PPR, the getter and metering valve are bypassed, which forfeits both cleaning of the gas by the getter and the convenience of flow rate adjustment with the metering valve.

In recirculation mode, *p*_stc_ is constant and the throughput is determined by the flow-restricting capillary (*C*_f_) and the metering valve. The 0.1 mm diameter, 1.2 cm long capillary limits the throughput to a maximum of 0.05 kPa·L/s at *p*_stc_ = 100 kPa. For PPR, the throughput is fixed by the combination of two flow-restricting capillaries (*C*_f_ and *C*_ppr_). In fill mode, the throughput is controlled by a combination of the regulator delivery pressure and the metering valve. The throughput is measured by closing valve V_oc_ (with V_1_ and V_2_ open) and observing the change in the capacitance manometer reading for a given period of time.

High gas purity is critical to maintaining a metastable population. Because of the high energy of the metastable state of ^3^He (20 eV), impurity atoms are ionized, resulting in destruction of the metastable. Gas purity in the OPCs is maintained using liquid nitrogen traps and a getter. The liquid nitrogen traps are simply two U-shaped tubes immersed in a 7 cm diameter, wide-mouth liquid nitrogen dewar. The getter is a commercial heated gas purifier [34], which we operate at a temperature of 570 K. Because the getter material is ferromagnetic, the gas is completely depolarized upon return to the optical pumping cell. (Because of the large surface area of getter material, depolarization would be expected even if the material were not ferromagnetic.) Given this depolarization, other sources of depolarization are acceptable in the return line, provided that they do not perturb the required uniformity of the magnetic field. The only other magnetic part is a stem in the metering valve, which is located sufficiently far from the polarized gas to avoid depolarization.

We have not studied the importance of the getter to gas purity. For PPR, (in which the getter is bypassed, but the liquid nitrogen traps are not) we have found that for a short term operation (hours), we do not see degradation of the gas purity. For long term operation, it should be possible to maintain high gas purity by using a getter line with a small conductance in parallel with the PPR line.

### 4.2 Optical Pumping Cell Preparation and Electrical Discharge

The OPCs were initially subject to the procedures that are typically used for the preparation of sealed cells: baking overnight at a temperature of 650 K, followed by several treatments with a strong rf discharge. When the apparatus is idle, impurities from the recirculation loop, especially the compressor, contaminate the surface of the OPCs. This results in low polarization when the discharge is first ignited, but on a time scale of 10 min to 30 min the discharge cleans the OPCs and the polarization increases.

Metastable ^3^He atoms are produced by a weak electrodeless rf discharge that is generated by coupling ≈1 W of power from a 13.6 MHz transmitter [35] to the cell using a resonant step-up transformer circuit. The circuit consists of a single-turn primary winding, and a 10-turn, 2 cm diameter secondary winding in parallel with a 110 pF, 4 kV, air-spaced variable capacitor [36] and electrodes on the cell. A separate loop of wire located near the transformer and connected to an oscilloscope provides convenient feedback when tuning the circuit. For small sealed cells, two electrodes encircle the ends of the cell. For the OPCs, four straight electrodes parallel to the length of the cell are connected with alternating polarity. Twin lead antenna cable mini-mizes stray capacitance in the connections to the electrodes. An antenna tuner [37] between the rf transmitter and the resonant circuit improves the impedance matching, and also provides a means for viewing the forward and reflected power.

The strong rf discharge required for cell cleaning was produced using a similar apparatus as described above, but in this case 100 W of rf power from a high power transmitter [38] is coupled to the cell by simply surrounding the cell with the step-up transformer windings. Due to the larger inductance from the increased diameter of the windings, the secondary winding was only a few turns.

### 4.3 Optical Pumping

The low pressure gas is polarized using an optical pumping apparatus similar to one that is described in detail elsewhere [14]. Here we review only the basic features, and point out details that are specific to this apparatus. The metastable atoms are optically pumped by 1083 nm wavelength light that is produced by a Nd:LMA (neodymium-doped lanthanum magnesium hexaluminate) laser. A Nd:LMA rod [39] was installed in a commercial arc-lamp-pumped Nd:YAG (neodymium-doped yttrium aluminum garnet) laser [40], and two temperature-controlled etalons were added to the laser cavity to tune the laser and narrow its bandwidth. We typically obtain 2 W to 3 W of laser light with a 2 GHz bandwidth.

The laser is tuned by maximizing the fluorescence signal from a ^3^He (or ^4^He) discharge cell that is illuminated by the weak beam of laser light transmitted through the high reflector at the back end of the laser cavity. The fluorescence emitted normal to the laser beam is detected by a large area photodiode [41]. A transmission window is created by the combination of a 1000 nm long-wave pass filter and the 1100 nm response cutoff of the silicon photodiode. The laser is tuned to the *C*_9_ line (2 ^3^*S*_1_ *F* = 3/2 → 2 ^3^*P*_0_ *F* = 1/2) for pure ^3^He, or to the ^4^He *D*_0_ line (2 ^3^*S*_1_ → 2 ^3^*P*_0_) for ^3^He-^4^He mixtures.

**Fig. 6:**
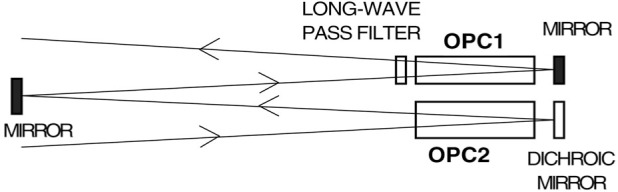
Schematic diagram of the arrangement used to illuminate both optical pumping cells. The scheme is described in Secs. 4.3 and 4.7.1.

To illuminate both OPCs we used the following arrangement: The diverging laser beam is circularly polarized before its diameter exceeds 2.5 cm, and then is expanded to a diameter of 6 cm using a telescope formed by two 7.5 cm diameter lenses with focal lengths of 10 cm and 30 cm. As shown in Fig. 6, the expanded beam traverses OPC2 and is reflected back with a slight deflection. Another steering mirror sends it through OPC1 and then the beam is similarly reflected back through OPC1. Since OPC1 is smaller in diameter than OPC2, the telescope is adjusted to produce a slowly converging beam. This scheme allows the full power of the laser to be incident on OPC2, and can be constructed without the use of large area polarizing optics. We used optically-sealed windows [42] to minimize distortion of the laser beam, but flame-sealed windows are adequate.

The discharge in each cell is controlled by individual rf power supplies, which allows for different discharge intensities and frequencies. In principle, a stronger discharge in OPC1 would improve the two-cell scheme [43], but in practice we find only weak sensitivity to the discharge strengths. The benefit from the two-cell arrangement is tested by extinguishing the discharge in OPC1, optimizing the discharge strength in OPC2, and observing the change in *P*_opc2_.

Because the optical pumping cells are located fairly far off the axis of the holding field coils, the relaxation time in the absence of the discharge is dominated by magnetic field gradients. For our typical operating pressures, we observe relaxation times of 200 s to 400 s. With the discharges on, the typical relaxation time is 70 s.

### 4.4 Compressor

There are several critical requirements on the compressor: 1) an inlet pressure on the order of 0.1 kPa at throughputs comparable to the polarizing rate for the metastable method; 2) sufficiently low leakage and permeation from the ambient atmosphere and outgassing of wetted compressor materials (ie., materials the gas contacts), 3) construction from non-magnetic materials so that the uniform magnetic field is not distorted, and 4) wetted materials that do not cause excessive depolarization during the time the gas is in the compressor. For the last requirement, the part of the compression cycle during which the surface to volume ratio is maximum is likely to be the most relevant [20].

With the above requirements as a guide, we chose a commercial two-stage diaphragm pump that is supplied without a motor [44]. A DC drive motor is located outside of the holding field coils and connects to the compressor via a 50 cm long brass shaft (visible on the right in Fig. 5). The gas contacts aluminum, Viton, and Teflon, which have reasonably low outgassing rates [31]. The largest surface areas in the pump are those of the aluminum pump heads and the Teflon-coated neoprene diaphragms. The one-way valves are Viton flaps. The pumps we assembled would often pass a helium leak test, and minimal use of high vacuum grease was always sufficient to eliminate any small leakage. By closing valves V_1_, V_2_, V_oc_, and V_cs_, and observing the slow rise in pressure on the capacitance manometer, the sum of outgassing and inward leakage was measured to be 2×10^‒4^ Pa·L/s (after many days under vacuum). All magnetic parts in the drive train were replaced with non-magnetic parts that were primarily made of Type 316 stainless steel [45] and annealed after machining. Type 316 stainless steel is one of most non-magnetic of the readily available stainless steels [46]. The wetted materials are non-magnetic and were expected to have sufficiently low relaxation rates [16,47]. Given the high surface to volume ratios present during compressor operation, this appears to be the most difficult requirement to satisfy, and is discussed further in Sec. 5.1.

The required modifications were primarily confined to the drive train of the compressor. The philosophy was not to redesign the pump, but instead to replace magnetic parts as directly as possible with non-magnetic equivalents. The modified compressor was assembled from new parts that were obtained from either the pump manufacturer or other commercial sources, or fabricated by the NIST shop. The drive train of the unmodified pump contains a steel shaft, eccentric, and counterweight; four steel radial ballbearings; three steel retaining rings; assorted steel screws; two aluminum connecting rods; thin steel spacers; and two steel diaphragm studs. The diaphragm is molded over the diaphragm stud, and screws into the connecting rod. Diaphragm studs were machined at NIST from Type 316 stainless steel and annealed to minimize residual magnetism before being sent to the pump manufacturer for incorporation into finished diaphragms. The shaft, eccentric, and counterweight were obtained in Type 316 stainless steel by special order to the pump manufacturer. The steel ballbearings were replaced with commercially available Type 316 stainless steel bearings, but for the main bearing a direct replacement was not available so a special sleeve was fabricated for the closest available bearing. For completeness, steel retaining rings were replaced by rings made from beryllium-copper. From a mechanical point of view, the most significant difference between the original pump and the modified pump is the bearings. The Type 316 bearings are not precision bearings and are much softer than typical bearing steel, resulting in a sloppier mechanical system. This does not seem to affect the achievable inlet pressure, but may affect the lifetime of the pump. Although the nominal rotation speed of the unmodified compressor is 1700 rpm, we typically operate the modified compressor at 800 rpm or less because the small improvement in inlet pressure obtained at higher speeds does not justify the increased stress, wear, and noise.

A magnetic permeability indicator [48] was used to test parts for magnetism. For all parts except the counterweight, there was no response at the most sensitive level (*μ* = 0.01). The small amount of magnetism observed in the counterweight was not expected to be a problem, and eventually this was confirmed by replacing it with a brass counterweight. Because the diaphragm studs are very close to the polarized gas, we checked that there was no observable change in the relaxation time of polarized gas at 0.13 kPa when the stud was located next to the cell.

Although the specification for the ultimate pressure (defined as the inlet pressure for zero gas flow) of the unmodified commercial compressor was 0.27 kPa, we obtained a typical ultimate pressure of 0.1 kPa, primarily by a simple modification of the orientation of the two pump heads: [49] The heads are normally oriented with the line between the inlet and outlet ports on each stage parallel to the motor-driven shaft. The ultimate pressure is substantially reduced if instead the heads are oriented so that the ports are in the plane normal to the shaft, and that the rotating shaft encounters the ports in the order that follows the gas flow, ie.: stage 1 inlet, stage 1 outlet, stage 2 inlet, stage 2 outlet. For the five modified pumps we have constructed, the ultimate pressures range between 0.08 kPa and 0.12 kPa.

The ultimate pressure increases linearly with the outlet pressure, except at the lowest outlet pressures. For our typical throughputs the inlet pressure is elevated. For the particular pump used for the work in this paper, the inlet pressure in kPa is approximately given by 0.013 + 0.67*p*_stc_ + 13*Q*, where *p*_stc_ is the StC (outlet) pressure in MPa, and *Q* is the throughput in units of kPa·L/s. In practice, the inlet pressure is typically between 0.1 kPa and 0.3 kPa, as discussed in Sec. 5.

The pressure between the two stages is typically 2.7 kPa at an outlet pressure of 100 kPa and is also proportional to the outlet pressure. However, it has little elevation at our typical throughputs because both stages have the same volume displacement, and the volume of gas entering the second stage is reduced by the compression ratio of the first stage.

We have limited data on the long term performance of the modified compressors. The first one was used for three years without significant difficulties, but the use was intermittent and the total operation time was on the order of 100 h, often at low speed. Once additional compressors were constructed, this compressor was operated continuously at somewhat higher speed for about one week until it failed due to a broken connecting rod. During operation a fine metallic powder was produced from the drive train, and upon being dismantled evidence for significant bearing wear was observed. Based on our limited experience to date, we consider the compressors suitable for intermittent use to fill cells with polarized gas, while reliability for long term continuous operation has not been addressed.

### 4.5 Storage Cells

For testing the apparatus, the storage cell need only have a sufficiently long relaxation time such that *f*_r_ is near unity. For *p*_stc_ = 100 kPa and a flow rate of 0.012 kPa·L/s, the average residence time in a 40 cm^3^ cell is only 0.1 h, so the typical relaxation time of a few hours obtained with Pyrex glass is adequate. For larger cells and applications, longer relaxation times are required. We have obtained relaxation times between 27 h and 72 h for cells constructed from aluminosilicate glasses (Corning 1720 [50] and GE180 [51]), which are known to yield long relaxation times because of their lower helium permeability [52]. Each cell was prepared by baking overnight at 650 K, with the compressor sealed off so as to obtain a typical base pressure of 4 × 10^−6^ Pa. Some discharge cleaning was performed, primarily to insure reasonably high polarization for the low pressure polarization measurement scheme described in Sec. 4.7.

Other requirements are imposed on the storage cells by the particular application for the polarized gas. We have developed cells for neutron spin filters and polarized gas MRI. Neutrons can be polarized (or analyzed) using polarized ^3^He because of the large spin dependence of the ^3^He absorption cross section. The large non-equilibrium ^3^He polarization produced by optical pumping allows for MRI of lungs and other body cavities. For both of these applications it is often convenient to detach the cell, for transport to a neutron beam line or MRI scanner. As shown in Figs. 4 and 5, a storage cell is equipped with glass valves (V_s_) and O-ring compression fittings (D), permitting the cell to be valved off and detached from the compression apparatus. To insure that the relaxation time of the storage cell is not compromised because of the glass valves, the valves are connected to the cell through glass capillaries (*C*_d_). At first, we chose the dimensions of the capillary to insure that the relaxation time due to diffusion through the capillary was always much greater than the cell relaxation time, assuming the worst case of complete depolarization of the ^3^He at the valve. In practice we have found that this requirement can be relaxed. For example, we have measured a relaxation time of 70 h in a 400 cm^3^ cell in which the diffusion time to the valve is only 3.3 h (5 cm long, 0.175 cm diameter capillary, cell pressure of 36 kPa). We have yet to see any additional relaxation from the valves, which would impart a pressure dependence to the storage cell relaxation time due to the pressure dependence of the diffusion time. The absence of any pressure dependence in the relaxation time for our typical range of storage cell pressures (35 kPa to 100 kPa) also indicates that relaxation due to magnetic field gradients [25] is negligible. Based on direct measurement of the relaxation time for a 7.2 cm ID, 10 cm long cell at pressures in the 0.1 kPa range, we estimate several thousand hours for the relaxation time due to magnetic field gradients.

Different construction techniques have been used for neutron spin filter cells as compared to cells for polarized gas MRI. The primary additional issue for the neutron application is the strong absorption of neutrons by ^10^B (absorption cross section of 3800 b at a neutron wavelength of 0.18 nm). Flat windows are also desirable so that the thickness of ^3^He is uniform across the neutron beam. Corning 1720 glass contains 5 % B_2_O_3_ [53], and natural abundance boron contains 20 % ^10^B. To avoid attenuation of the neutron beam, optical quality windows were constructed from a special batch of Corning 1720 glass [50] that was manufactured using B_2_O_3_ with an isotopic composition of 99.62 % ^11^B [54]. For a neutron beam with a 10 % spread in wavelength we measured the absorption through a cell with two 4.1 mm thick windows to be 0.80, 0.79, and 0.66 at wavelengths of 0.5 nm, 1.0 nm, and 2.0 nm, respectively. The corresponding transmissions that are expected, assuming loss from the small amount of ^10^B in this glass only, are estimated to be 0.93, 0.87, and 0.75. (The absorption due to the ^10^B increases linearly with increasing neutron wavelength because of the wavelength dependence of the cross section.) The additional observed loss of about 10 % is consistent with typical neutron scattering from glasses. Spin filter cells identified as “Mercury” (4.4 cm ID, 10 cm length), “Neptune” (7.2 cm ID, 10 cm length), and “Saturn” (9.5 cm ID, 15 cm length) were each constructed using optical seals of such windows to a cylindrical body of normal Corning 1720. The relaxation times for these three cells just after preparation were 37 h, 72 h, and 50 h, respectively. Results with these cells are discussed further below.

GE180 glass is boron-free, but is only commercially available in narrow diameter tubing. It is possible to attach windows of GE180 glass to a body of Corning 1720. We have constructed two such 5.9 cm diameter cells, one with flame-sealed rounded windows and another with optically sealed windows, and obtained relaxation times of 27 h and 25 h, respectively. We have tested one 40 cm^3^ cell constructed entirely of GE180 glass and obtained a relaxation time of only 6 h. Although this value was unexpectedly low, we do not draw any general conclusions about GE180 cells from this one test.

For application to polarized gas MRI, 250 cm^3^ cells are filled to 100 kPa while being maintained at 77 K (see Sec. 4.6), resulting in a pressure of 400 kPa at 300 K. To accommodate this pressure we use a rounded-end construction, and threaded glass valves rather than stopcock valves. Our first cell for this application was constructed from Corning 7052 glass [23], and only yielded a initial relaxation time of 15 h, which subsequently declined to 7 h. A cell from normal Corning 1720 was then constructed and yielded a relaxation time of 33 h.

A few comments will put our cell relaxation times in context. The surfaces of cells for spin-exchange optical pumping are inherently modified by the presence of rubidium, whereas the surfaces of storage cells for metastability-exchange may be bare glass, or deliberately coated with metal. Relaxation times over 200 h, limited by magnetic dipolar spin relaxation, have been obtained in cells for spin-exchange optical pumping [52,55,56]. It has been shown that the relaxation time of a metal-coated glass cell can be substantially longer than would be obtained with uncoated glass [57], so the rubidium plays an important role in the relaxation times for spin-exchange cells. Other evidence for this statement includes 1) a relaxation time of over 250 h obtained with a spin-exchange cell made from Corning 7056 glass [55], as compared to 10 h for uncoated 7056 cells [16], and 2) a relaxation time of 176 h obtained with a Cs-coated quartz cell [2], as compared to ~1 h for uncoated quartz cells [52]. Since our cells are un-coated, the most appropriate comparison is with other uncoated cells. Uncoated cells for metastability-exchange may be either sealed low pressure cells or refillable cells for use with a compression apparatus. Better control of impurities is possible for sealed cells. Direct measurement of relaxation times for sealed, uncoated low pressure cells include values between 5 h and 56 h [16,52,57]. Values as high as 140 h, potentially limited by magnetic field gradients, have been observed for the transverse relaxation time *T*_2_ in sealed low pressure cells [58]. Prior to the work in this paper, the only results with refillable cells have been from the Mainz and ILL groups. Most of their work has been with coated cells [2,57], but a relaxation time of 75 h was reported for a cell made from iron-free Supremax glass [2,59], and 30 h for cells made from Corning 1720 [60].

Given this context, the observed range of 25 h to 70 h for the relaxation times of our storage cells seems reasonable; indeed it is encouraging that these values can be maintained in the less than pristine environment of the diaphragm compressor. As for the stability of these relaxation times, we do see variations, but not necessarily with any clear pattern. Variations sometimes occur when the gas is recirculated, or if it is evacuated and then refilled. We presume that these variations are due to changes in impurities on the surface of the cell. If a cell exhibits a drop in relaxation time, recirculating the gas will sometimes help. We have the greatest amount of data with the cell Mercury: In the first few months of use, we observed relaxation times between 27 h and 37 h. After sitting on the shelf for about a year, a capillary on Mercury was accidentally broken. After being repaired, it was reinstalled on the apparatus; weak discharging was done to permit a new calibration (see Sec. 4.7), but it was not baked. The relaxation time was measured to be 29 h, within the range of the earlier measurements. However, in the course of subsequent experiments, the relaxation time dropped to 16 h for no clear reason, and did not recover after baking. After cleaning with a weak discharge, the relaxation time did recover to 26 h, and has remained between 26 h and 32 h thereafter. For our other cells, we have less history: the relaxation time of the cell Neptune (Saturn) has varied between 40 h and 72 h (32 h and 66 h).

### 4.6 Storage Cell Cooling

Cryogenic methods have been used to increase the density of polarized ^3^He gas [61,62]. We have employed a liquid nitrogen bath to roughly quadruple the density of the gas for the same storage cell pressure, and thus obtain correspondingly higher pressure when the cell is returned to room temperature. Using this method, we obtained a room temperature pressure of 400 kPa, which was convenient for gas delivery in a polarized gas MRI demonstration [23]. Alternatively, operation at lower storage cell pressure for a given density permits higher values of *P*_stc_ because of the increase in *f*_c_ (see Sec. 5). This approach was used for tests of a neutron spin filter [1]. Our space constraints led us to construct custom size containers from solid blocks of styrofoam. Unfortunately, the particular styrofoam that was available was found to be porous, so we coated the inside of these containers with an epoxy with good cryogenic properties [63].

A potential problem in employing this approach is the effect of cooling on the relaxation time of the cell. For Pyrex, the competing effects of decreased permeation and increased sticking time lead to a maximum in the relaxation time at 125 K, and at 77 K values comparable to those obtained at room temperature can be obtained [52]. For aluminosilicate cells, the sticking time dominates, so the relaxation time generally decreases with decreasing temperature. Nevertheless, the relaxation times for aluminosilicate cells are generally high enough that the decrease does not adversely affect the achievable polarization for a cell at liquid nitrogen temperature. For example, we have observed a decrease from **τ**_stc_ = 30 h at 300 K to **τ**_stc_ = 15 h at 77 K in the cell Mercury. This result is given as an example, not as a definitive statement for aluminosilicate cells in general.

We have experienced one unresolved difficulty with this cooling approach. Even after the cell has warmed to room temperature, the relaxation time does not return to its room temperature value. The drop in relaxation time is not irreversible, and often the original room temperature value can be recovered by simply recirculating the gas. We presume that this drop in relaxation time is due to impurities that are weakly bound to the cell walls. These results make it unclear what fraction of the observed decrease in relaxation time upon cooling is truly due to temperature dependence. Some improvement in the recovery of the relaxation time has been obtained by using a liquid nitrogen trap between the compressor and the storage cell (not shown in Fig. 4).

### 4.7 Polarization Measurement

The polarization in the OPCs is determined by measuring the degree of circular polarization of the 668 nm light emitted by the discharge [64,65]. The polarization in the StC is determined by NMR measurements, which are calibrated by optically pumping low-pressure gas in the StC directly and recording both the optical and NMR signals. This method eliminates the need for painstaking water-based calibrations. Although much smaller than the signal from compressed gas, the signal from low pressure gas is still typically 30 times larger than a water signal at our typical magnetic field of 2.6 mT. NMR is also used to determine cell relaxation times.

#### 4.7.1 Optical Polarimetry

For accurate polarimetry using the 668 nm emission line a dichroic mirror that reflects laser light but does not reflect 668 nm light is used at the back of OPC2 (see Fig. 6). For OPC1, an ordinary mirror is used at the back, and a long-wave pass filter is located in front so that the polarimeter only views OPC2. The goal of both wavelength selection schemes is to reduce the small probability of viewing 668 nm wavelength light that may have had its polarization altered upon reflection. When measuring the polarization in OPC1 is desired, the filter is moved behind OPC1 and another filter is located in front of OPC2.

Employing the optical method requires knowledge of the pressure-dependent ratio of the nuclear polarization to the polarization of the light, which varies between 7 and 13 in the pressure range between 0.05 kPa and 0.5 kPa. Two calibrations of this ratio, one linked to NMR measurements of a water sample [66] and another linked to measurements of pump light absorption [67], each have typical relative standard uncertainties of 2.5 % and agree within those uncertainties. Typical sources of uncertainty in the application of this method are described in these two calibrations. An additional systematic error relevant to a flowing system would arise from a pressure drop between the OPCs and the capacitance manometer. From the calculated conductance of the relevant tubing, we estimate the pressure could be higher in OPC2 by no more than 0.03 kPa (at a throughput of 0.015 kPa·L/s) which would result in no more than a 3 % underestimate in the measurement of *P*_opc2_. Overall we estimate that the total relative standard uncertainty in our routine optical polarimetry is 4 %.

Calibration of the ratio for mixtures are less accurate and less extensive. For a mixture of 25 % ^3^He and 75 % ^4^He the ratio has been found to scale with the inverse of the percentage of ^3^He, with a relative uncertainty of 5 % to 10 % [13]. This result is expected to be generally applicable because only the emission from ^3^He is polarized. The vendor’s specified relative uncertainty of 5 % in the percentage of ^3^He also contributes to the total relative standard uncertainty in the calibration of the optical method for mixtures. Better knowledge of the gas mixture can be obtained by gas analysis, but the mixtures purchased were cost-effective for routine use. In addition, the uncertainty in application of the method to mixtures is increased because of the decreased size of the optical signal. Overall we estimate that the total relative standard uncertainty in our routine optical polarimetry for mixtures is 8 %.

#### 4.7.2 NMR Polarimetry

**Fig. 7:**
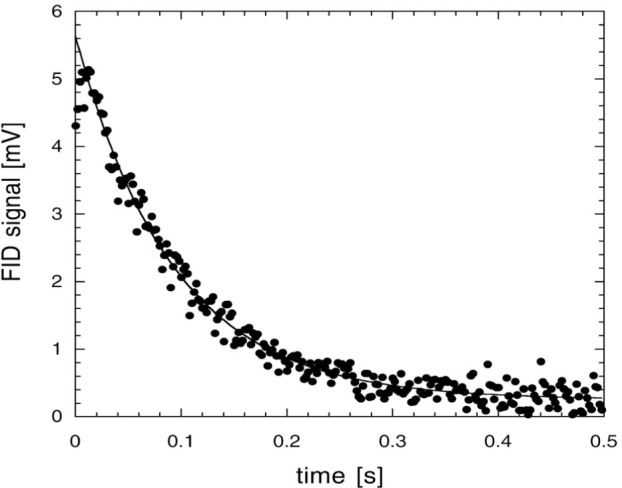
Free induction decay NMR signal from the cell Neptune, using a fully destructive π/2 rad tip angle. The cell was filled to a pressure of 0.13 kPa of pure ^3^He and polarized to 45 % by direct optical pumping. The first few data points are low because of the 3 ms lock-in time constant. The solid line shows a fit to an exponential decay for times greater than 0.016 s.

**Fig. 8:**
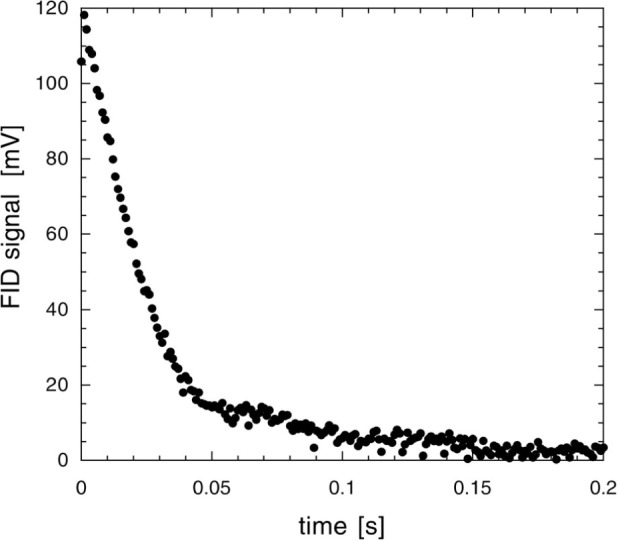
Free induction decay NMR signal from the cell Neptune, using a tip angle of 0.070 rad. Using the compression apparatus, the cell was filled to a pressure of 100 kPa of a gas mixture (33 % ^3^He, 67 % ^4^He). The first point is low because of the 0.3 ms lock-in time constant. The shape of the FID is not exponential, but instead is given by the Fourier transform of the spectral distribution of Larmor frequencies in the cell. Comparing to the data in Fig. 7, and accounting for the mixture ratio for the compressed gas and the different pressures and tip angles leads to *P*_stc_ = 0.51.

Free induction decay (FID) [68,69] is used to measure the polarization of the compressed gas in the storage cell. In this method, the magnetization is tipped away from the quantization axis by applying a short pulse of radiation at the Larmor frequency (85 kHz for our typical operating field of 2.6 mT). After this pulse, the transverse component of magnetization freely precesses around the quantization axis at the Larmor frequency while decaying in magnitude. The time scale of the decay, *T*_2_, is related to dephasing of the individual nuclear spins due to inhomogeneity in the static magnetic field. The initial size of the signal is proportional to the magnetization. The free precession is detected with a resonant pickup coil, connected to a dual-phase lock-in amplifier. By calibrating the response of the pickup coil using optical polarimetry at a known low pressure, and measuring the pressure in the storage cell, the storage cell polarization is determined. Figure 7 shows an FID signal from 45 % polarized gas produced by direct optical pumping of pure ^3^He at a pressure of 0.13 kPa, which we will refer to as the calibration FID signal. Figure 8 shows an FID signal from a compressed gas mixture (33 % ^3^He, 67 % ^4^He) at a pressure of 100 kPa. Whereas a fully destructive π/2 rad tip angle was used for the calibration FID signal, a tip angle of 0.070 rad was used for the compressed gas. Accounting for the different pressures and tip angles, and the mixture ratio for the compressed gas, leads to *P*_stc_ = 0.51 for the data in Fig. 8. Although performing the optical calibration of the NMR system with the same mixture ratio as the compressed gas cancels out inaccuracy in the knowledge of the mixture ratio, we used pure ^3^He for the calibration FID signal because it yields both a larger FID signal and a larger optical signal.

The initial tip angle of the magnetization is controlled by the amplitude of the rf pulse applied to the NMR drive coils. The rf amplitude required for a/2 tip angle is determined by two methods: 1) by observing complete loss of the optical signal from low pressure gas after the rf pulse, 2) by observing complete loss of the FID signal from compressed gas after the rf pulse. In the low pressure case, the discharge is extinguished during NMR, and reignited after the rf pulse to measure the remaining optical signal. In the compressed gas case, a second π/2 tip is performed to measure the remaining NMR signal. Both measurements are typically performed, allowing a double check on the tip angle. A variation on the NMR test is to determine the required amplitude for a π pulse by minimizing the remaining signal after such a pulse.

By using relatively small tip angles, the polarization is monitored with minimal loss of polarization. The required amplitude is determined by attenuation relative to the value needed for a π/2 tip, and is also checked by performing a sequence of several small tips and measuring the remaining polarization. For a tip angle of 0.17 rad, the signal height (as compared to a π/2 tip) is reduced by a factor of sin(0.17) = 0.169, but the fraction of the initial polarization remaining after the tip is cos(0.17) = 0.986.

The temporal behavior of the FID is not important as long it does not affect the measurement of the initial size of the signal. For the compressed gas, the shape of the FID is given by the Fourier transform of the spectral distribution of Larmor frequencies in the cell. Since we do not generally know the functional form of the shape of the FID, the initial size of the signal is not extracted by a fitting procedure, but rather is determined directly from the first data points of the FID (ignoring the points that are initially low because of the lock-in time constant). If *T*_2_ is too short, determining the initial size becomes more difficult because shorter lock-in time constants are required. *T*_2_ is quite sensitive to magnetic field inhomogeneity, so we found that it is important to locate the storage cell close to the center of the holding field coils. *T*_2_ typically ranged between 100 ms for small cells down to 20 ms for large cells. For the relatively large signals from compressed gas, we could use corresponding lock-in time constants between 3 ms and 0.3 ms. For the low pressure gas, the long mean free path allows for “motional narrowing” [70], which leads to an exponential shape for the calibration FID signal, as shown in Fig. 7. Hence for the calibration FID signal, we determined the initial size of the signal from a fit of the FID data to an exponential decay. Motional narrowing also leads to an increase in *T*_2_ because of averaging of the gradient, which is convenient because longer lock-in time constants are desirable when measuring the smaller signal from low pressure gas. Although this increase was observed for small cells, resulting in *T*_2_ ≈ 1 s, the increase was less pronounced for larger cells. We attribute this effect to diffusion of gas on the time scale of the FID. The gas near the edge of a large cell, where the gradient may be larger, may only contribute a small amount to the pickup coil signal for the compressed gas, while diffusion at low pressure results in a larger range of gradients sampled by the gas contributing to the pickup coil signal.

For a sufficiently large magnetization and a pickup coil with a high quality factor, FID can be influenced by radiation damping [68,71] ie. the effect on the magnetization from the currents induced in the pickup coil. These currents always act upon the magnetization so as to drive it towards the lower energy nuclear magnetic substate. If the nuclei are initially in the lower (higher) energy state and the magnetization is tipped by an angle less than π/2, the currents generated in the pickup coil shorten (lengthen) *T*_2_ and result in a value for the remaining longitudinal magnetization that is larger (smaller) than the cosine of the tip angle. Whereas in the lower energy case *T*_2_ is simply shortened, in the higher energy case the shape of the FID can be dramatically modified because the transverse magnetization can increase during the free precession. Here we are concerned with the possible effect of radiation damping on FID measurements, in particular on the determination of *P*_stc_, but also on the measurement of cell relaxation times. In general, the initial size of the FID signal is unchanged. However, the effect on the remaining magnetization can influence the determination of the required amplitude for a π/2 tip by the method discussed above. In this case, the alternative method of determining the amplitude for a π tip proves to be less sensitive to radiation damping (see Appendix A). To keep radiation damping effects small, we deliberately decreased the quality factor of the pickup coil from 120 to 30 by adding two 1.3 kΩ resistors to the pickup coil circuit. In addition we optically pumped to the lower energy state. More discussion of the effects of radiation damping can be found in the Appendix.

A tip angle of π/2 is obtained from a 5 ms long, 6 V peak amplitude signal applied to a square Helmholtz pair, 23.8 cm on a side. Each coil has 11 turns of 0.41 mm diameter wire, and the magnitude of the impedance was measured to be 60 Ω. Since the impedance is dominated by the inductive reactance, these data yield 0.22 mH for the inductance, close to the calculated value of 0.23 mH [72]. The separation of the two coils is close to 0.5445 times the length of one of the sides, which is the ideal separation for a square Helmholtz pair [73,74]. The drive coil is energized using a reed relay with specified operate and bounce times of 0.62 ms and 0.40 ms, respectively.

The square pickup coil has 400 turns of 0.20 mm diameter wire and is 6.6 cm on a side, and 0.50 cm on a side of the wire cross section. Since the NMR signal is directly proportional to frequency, we aimed for a resonant frequency near 100 kHz, the maximum reference frequency for the lock-in amplifier. Stray capacitance was minimized by using a preamplifier that was located only 60 cm from the pickup coil (the primary reason it was not even closer was due to its magnetic housing). The connection to the preamplifier was 30 cm of RG58 coaxial cable and 25 cm of RG62 coaxial cable. RG62 has lower capacitance than RG58, but is slightly magnetic, so it was not used in close proximity to the cell. Based on a calculated value of 22 mH for the inductance of the pickup coil and the measured resonant frequency of 85 kHz, the total capacitance is estimated to be 160 pF. To prevent overloading the preamplifier from pickup of the drive coil pulse, the inputs are kept shorted by a reed relay until 1 ms after the drive coil pulse ends. The relay, as well as protection diodes and the aforementioned resistors are located in a small box just before the preamplifier.

The scatter in the data in Figs. 7 and 8 is due to electrical noise on the pickup coil signal. To improve the accuracy of the calibration against optical polarimetry, several measurements of the relatively small calibration FID signal are averaged. The signal to noise ratio depends on the size of the cell, the distance between the cell and the pickup coil (which is larger when the cell dewar is used), the polarization and gas pressure, and the noise level on the particular day that the calibration FID signals are obtained. For the data discussed in this paper, we estimate the typical relative standard uncertainty in determining the initial size of the calibration FID signal to be 5 %. The total relative standard uncertainty in measurements of *P*_stc_ is estimated to be 9 %, which includes additional contributions from the optical polarimetry itself (4 %, see Sec. 4.7.1), the initial size of the FID signal from the compressed gas (3 %), the ratios of the tip angles and gas pressures for the two FID signals (3 % each), and the mixture ratio (5 % for compression of mixtures).

## 5. Results

### 5.1 Polarization Preservation During Compression

**Fig. 9:**
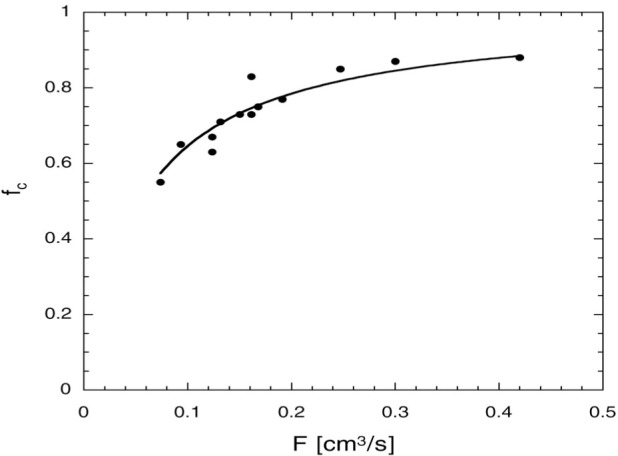
The variation of *f*_c_ with *F*. The data are shown by solid circles, along with a fit to Eq. (5).

For this apparatus, the fraction of polarization preserved during compression is clearly the most important parameter to characterize. Recirculation mode is convenient for such tests because the optical pumping cell pressure and polarization, the storage cell pressure and polarization, and the throughput can be measured under equilibrium conditions. We find that *f*_c_ increases with the volume flow rate at the outlet of the second stage of the compressor, *F* (*F* = *Q*/*p*_stc_). This is reasonable because the time the gas spends in the second stage of the compressor is inversely proportional to the volume flow rate. The volume flow rate is the slowest at the outlet of the second stage because for a given throughput, the volume flow rate at any point is inversely proportional to the pressure at that point. Figure 9 shows the variation of *f*_c_ with *F*. Although values of *f*_c_ approaching 0.9 are observed at high volume flow rates, values of 0.6 to 0.8 are more typical. The volume flow rate is optimized by trading off the increase in *f*_c_ against the decrease in *P*_opc_. Experimentally *F* is determined by measuring the ratio of the throughput to the storage cell pressure. The value of *f*_c_ was determined from the measured values of *P*_stc_ and *P*_opc2_, and corrected slightly by the value of *f*_r_. These data were acquired using a 40 cm^3^ Pyrex cell, with a relaxation time of 2.7 h. The value of *f*_r_ varied between 0.94 and 1, so the correction was small.

The identification of the second stage of the compressor as the likely source of the decrease in *f*_c_ deserves further comment. The pressure at other points in the compressor also depends on both the storage cell pressure and the throughput (see Sec. 4.4), so one might speculate that the behavior of *f*_c_ is related to changes in the time spent at these other locations. For example, the gas moves rapidly at low pressure, but is much more sensitive to magnetic field gradients. However, we have not seen any clear correlation with the optical pumping cell pressure, which would be an indicator of a gradient issue. As for the pressure between the stages, it is almost independent of throughput, and *f*_c_ most definitely is not. Further evidence to support the claim that the second stage is the dominant source of relaxation during compression was obtained by reconfiguring the apparatus so that the polarization could be measured in a buffer cell between the stages. For values of *F* at the lower end of the data shown in Fig. 9, the polarization between the two stages was still 90 % of *P*_opc2_.

We attempted to decrease the relaxation in the second stage by controlling the transfer of gas from the first stage to the second stage. A pneumatic, non-magnetic valve was installed on the inlet port of the second stage. The idea was to build up pressure in the buffer cell and periodically open the valve so that the released gas would travel through the second stage more quickly. (The optical pumping cell pressure would also vary proportionally with the buffer cell pressure.) For reasons that are not completely understood, any improvement in *f*_c_ was minor, at best. The lack of improvement may be due to increased relaxation of the gas residing in the second stage of the compressor while the valve is closed.

We do not fully understand the relaxation observed during compression, but speculate that it is due to the large surface to volume ratio in the compressor head. The total surface area of the Teflon-coated diaphragm and aluminum head plate is *S* = 60 cm^2^, and the volume of the compressor head varies between 10 cm^3^ and essentially 0 cm^3^. At our typical flow rates, the gas spends many compression cycles in the head. Although the exact details of the volume as a function of time are not known, assuming a simple sinusoidal dependence leads to **τ**_c_ = (*αV*_a_)/(*S***κ**^1/2^), where **τ**_c_ is the relaxation time in the compressor head, *V*_a_ is the average volume of the head, *α* is the relaxation time for *S*/*V*_a_ = 1 cm^−1^ , and ***κ*** is the compression ratio. Using this result, *f*_c_ is given by

$$f_{c}=\frac{1}{1+[(2S)/(\alpha F\kappa^{1/2})]}.$$ (5)

Figure 9 shows a fit of the data for *f*_c_ vs *F* to this form, with *S* = 60 cm^2^ and *κ* = 38, and a fitted parameter. A value of *α* = 360 s/cm was extracted from the fit, where we have yet to distinguish between the presumably different values for *α* for aluminum and Teflon. There are few relaxation time measurements in the literature, and we have not measured the relaxation times for the Teflon coated diaphragm or the aluminum head. Potentially relevant relaxation time values from the literature include: 14 min for Viton and 10 h for aluminum (surface to volume ratio near 1 cm^−1^) [47] and 24 min for “utility sheet aluminum” and 17 h for spectrographically pure aluminum (surface to volume ratio of 0.08 cm^−1^) [16]. (We considered Viton to be relevant only because it might be similar to Teflon.) Hence a value of 360 s for a surface to volume ratio of 1 cm^−1^ seems somewhat short, but without doing our own controlled measurements for the particular Teflon and aluminum used in the compressor, not much can be concluded. In this case it would be appropriate to test the materials as used, in contrast with the stringent cleaning procedures used in previous studies [16]. We found that coating the Teflon diaphragm with aluminum had no discernable effect on *f*_c_, which either indicates that the Teflon does not dominate the relaxation, or that the hypothesis that the behavior of *f*_c_ is related to relaxation on materials in the compressor head is invalid. Based on the work to date, we do not have a definitive understanding of the relaxation, but based on the analysis above the value for that is required to explain the behavior is not unreasonable.

Whereas *f*_c_ is fixed for operation in recirculation mode, it varies during operation in fill mode. In general, a higher polarization is obtained when filling a cell to a given storage cell pressure as compared to recirculating gas at the same storage cell pressure. This is expected because for a given throughput, *p*_stc_ rises linearly from zero to the value that it ultimately has for recirculation mode, hence the average value of *F* (hence *f*_c_) is higher. Alternatively, if the throughput is varied during the fill so as to keep a constant value for *F*, the conditions are improved for the optical pumping because the average value of *t*_opc_ is larger. In either case, the average value of *P*_0_ is higher because the average optical pumping cell pressure is lower.

### 5.2 Optical Pumping Cell Polarization

Given the need for high volume flow rates in order to preserve the polarization during compression, efficient optical pumping is desirable. In this section we provide a sample comparison of the values of optical pumping cell polarization that we obtain with gas flow (*P*_opc1_ and *P*_opc2_), as compared to the values without flow (*P*_0_).

To compare the measured value of *P*_opc2_ during compressor operation to the expected value based on Eq. (4) requires determination of the mean residence times *t*_opc1_ and *t*_opc2_, and the four optical pumping parameters *P*_0_, *f*, **τ**_1_, and **τ**_2_. The residence times were determined by shuttering the laser light and measuring the resultant exponential decline in polarization as the polarized gas was replaced by unpolarized gas. The residence times obtained were consistent with those determined from throughput and pressure measurements. A small correction (10 %) is applied to account for the relaxation in the discharge. The optical pumping parameters cannot be measured by simply stopping the gas flow because the pressures in the OPCs change when this is done. Instead we measured the rise in polarization when the laser illuminated each OPC, which were maintained at the pressure and discharge intensity used during compression. For example, for *p*_stc_ = 80 kPa and *Q* = 0.015 kPa·L/s, the values of *p*_opc1_ and *p*_opc2_ are 0.47 kPa and 0.27 kPa, respectively, and we measured *f P*_0_ = 0.50, *P*_0_ = 0.61, 1 = 5.2 s, and 2 = 5.1 s (33 % ^3^He, 67 % ^4^He mixture). For this throughput, *t*_opc1_ and *t*_opc2_ are 11.0 s and 9.9 s, respectively. For optical pumping of OPC2 or OPC1 only, Eq. (3) yields *P*_opc2_ = 0.40 and *P*_opc1_ = 0.34, slightly lower than the observed values of 0.44 and 0.36. For the diffusion-restricted double cell case, Eq. (4) yields *P*_opc2_ = 0.52, whereas 0.53 was observed.

Fitting the rise in polarization with an exponential is only an approximation that yields some kind of average time constant. We also used a numerical calculation to include the known non-exponential behavior of metastability-exchange optical pumping, which has been characterized as a dependence of the effective time constant on the equilibrium polarization in the cell [14]. We incorporated a linear decline in with increasing polarization [14] into the numerical calculation. The values of the optical pumping parameters in the numerical calculation were adjusted to reproduce the results of the static optical pumping tests. These parameters were then used in a numerical calculation that included gas flow. For optical pumping of OPC2 or OPC1 only, the numerical calculation yielded *P*_opc2_ = 0.39 or *P*_opc1_ = 0.33, and for the diffusion-restricted double cell case, *P*_opc2_ = 0.50 was obtained. As expected, these values are lower than those obtained from Eq. (3) and Eq. (4) above, which increases the discrepancy between the calculated and measured results for optical pumping with gas flow.

Obtaining slightly higher polarization than the calculated result was surprising. We checked the measured values of the optical pumping parameters for OPC2 by optically pumping a sealed cell of the same dimensions, and obtained similar values. (This test also indicates that impurities do not significantly affect the achievable polarization in the OPCs, at least for our relatively strong discharge intensities.) As a further test, we also measured the time constants for the rise in the polarization of the flowing gas upon illuminating each cell, denoted by **τ**_2f_ and **τ**_1f_. **τ**_2_ was determined using the relationship $\tau_{2}^{-1}=\tau_{2f}^{-1}-t_{\text{opc}2}^{-1}$, and similarly for **τ**_1_. For reasons that are not understood, **τ**_2_ determined from these flowing gas measurements was half of the value determined from the static measurements, while better agreement was found for **τ**_1_. These results would reduce the discrepancy discussed above, but we do not have an explanation for the apparent difference in the time constants extracted from static and flowing measurements. Hence it is unclear whether we can claim accurate modelling of the optical pumping, and it appears that the achievable polarization with gas flow is higher than one might expect from our modelling. Nevertheless, the diffusion restricted double cell arrangement clearly yields higher values for *P*_opc2_, and there is reasonable agreement in the relative improvement as compared to the simple single cell.

By employing PPR, the value of *P*_opc2_ was increased to 0.58. The value of the polarization of the returning gas can be determined by shuttering the laser light to the OPCs and waiting long enough for the gas in the OPCs to be replaced by gas returning from the storage cell. The optical pumping cell polarization slowly drops as the freshly polarized gas exits, and then stabilizes at the value for the polarization of the returning gas. Typically we find that *P*_opc2_ increases to about 85 % of *P*_stc_.

### 5.3 Results for Applications

In this section we present our current results for two applications: cold neutron spin filters and polarized gas MRI. We focus here on the capability of the compressor to produce polarized gas for these applications, rather than the applications themselves.

For a neutron spin filter operating in a given range of neutron energies, the product of storage cell pressure and cell length is chosen based on a trade-off between high neutron polarization (or analyzing power) and high transmission. For cold neutrons, a desirable product is typically 400 kPa·cm. As discussed above, we obtain the highest ^3^He polarization by using ^3^He-^4^He mixtures and minimizing the storage cell pressure. Although using pure ^3^He would result in the lowest storage cell pressure, the increase in the achievable optical pumping cell polarization using mixtures proves to be more important. The combination of these two requirements makes long cells preferable, but for practical considerations we have limited the cell length to a maximum of 15 cm. Using the cell Neptune (10 cm long), we have obtained *P*_stc_ = 0.33 for a mixture of 50 % ^3^He and 50 % ^4^He continuously recirculated at *p*_stc_ = 80 kPa. Other operating parameters for this test were: *p*_opc2_ = 0.26 kPa, *Q* = 0.016 kPa·L/s, *P*_opc2_ = 0.45, and *f*_c_ = 0.74. Using PPR, *P*_opc2_ increased to 0.49 and *P*_stc_ to 0.37, and in fill mode we obtained *P*_stc_ = 0.45.

If the cell length were increased to 15 cm, a mixture of 33 % ^3^He and 66 % ^4^He could be used for the same value of *p*_stc_. For this mixture ratio and almost the same values for *p*_opc2_, *Q*, and *f*_c_, we obtained *P*_stc_ = 0.40 and *P*_opc2_ = 0.54. For PPR, *P*_opc2_ increased to 0.58 and *P*_stc_ to 0.43. Filling a cell to 100 kPa yielded *P*_stc_ = 0.52. Three hours after this particular fill of Neptune, *P*_stc_ was determined to be 0.48 by neutron transmission methods [1], which is consistent with the relaxation time of the cell.

For polarized gas MRI it is desirable to either maxi-mize the total magnetization, or to obtain a given magnetization for the least amount of ^3^He consumed. Depending on the future of this field, it may also prove desirable to produce polarized ^3^He rapidly. In our previously reported application to polarized gas MRI, we produced 100 kPa·L of 15 % polarized ^3^He gas in 3 h. The 250 cm^3^ cell was filled to a pressure of 100 kPa at liquid nitrogen temperature, yielding a room temperature pressure of 400 kPa. We now consider ^3^He-^4^He mixtures to be the most efficient approach to applying this apparatus for polarized gas MRI. Use of a 50 % ^3^He and 50 % ^4^He mixture should yield *P*_stc_ ≈ 0.44, ie. an effective overall gas polarization of 0.22. If an adequate signal to noise can be obtained with a lower magnetization, the fraction of ^3^He can be decreased.

## 6. Conclusion

We have described an apparatus that demonstrates that highly polarized ^3^He gas, produced at a typical pressure of 0.25 kPa by metastability-exchange optical pumping, can be compressed to a pressure of 100 kPa using a compact, simple, inexpensive apparatus that is based on a modified commercial diaphragm pump. For typical operating conditions, the apparatus can preserve about 75 % of the polarization produced by optical pumping at low pressure. The loss of polarization during compression decreases with increasing volume flow rate, necessitating flow rates comparable to the optical pumping rate. To obtain the highest polarization of the low pressure gas in a compact system operated at such flow rates, we have exploited two schemes: 1) a two-cell arrangement with a diffusion restriction, and 2) polarization preserving recirculation. These techniques allow us to obtain 85 % to 95 % of the achievable optical pumping cell polarization in the absence of gas flow (*P*_0_), but the discharge intensity required for rapid optical pumping limits *P*_0_. For typical operating conditions, the optical pumping cell pressure of 0.25 kPa is higher than optimum for pure ^3^He gas, and limits the achievable polarization. This issue is mitigated, but not completely eliminated, by the use of ^3^He-^4^He mixtures; despite operation at higher overall pressure and flow rate, the best results are obtained with mixtures. In general “fill mode” yields better results than “recirculation mode”. All of these issues yield an apparatus for which the performance is strongly dependent on the required storage cell pressure of polarized ^3^He but is fairly independent of the size, shape, and relaxation time of the storage cell. Presently we have obtained as high as 52 % ^3^He polarization for a storage cell filled to 100 kPa of a mixture containing 33 % ^3^He and 67 % ^4^He.

The apparatus is presently being applied for neutron spin filters. Given that we can obtain acceptable ^3^He polarization for a cold neutron spin filter, the key issue now is longer relaxation time cells. The relaxation times for our present cells are more than adequate for a continuously recirculated spin filter, but it is currently more convenient to fill cells and transport them to the neutron beam line. We have recently obtained a 200 h relaxation time using Rb-coated GE180 glass [78], and in a first test this long lifetime was not significantly degraded after cycling to liquid nitrogen temperature.

For polarized gas MRI applications, a liquid nitrogen cooling bath provides a convenient method for increasing the final cell pressure. Based on our tests, we expect that the apparatus can produce 100 kPa·L of 20 % polarized ^3^He gas in 2 hours using optical pumping of either pure ^3^He gas or a ^3^He-^4^He mixture. Higher production rates are clearly possible at the expense of the final polarization.

The ultimate goal is to realize the full potential of the MEOP method with a compact device. The key issue is reducing the polarization loss in the compressor, not just for the obvious reasons but also because of the impact on the utility of polarization preserving recirculation. If lossless compression is combined with PPR, the potential increase in polarization from each pass through the optical pumping cells would not be diminished by recovering the loss in each previous pass through the compressor. Hence the asymptotic approach to *P*_0_ can be distributed over several passes through the recirculation loop, rather than being required to occur in a single pass. This would decrease the need for large optical pumping cells and strong discharge intensities. In addition, this scheme would be more tolerant of higher flow rates, thus increasing the chance that the presumed lossless compression can actually be attained. Assuming that gas purity does not limit *P*_0_, lossless compression combined with PPR should allow storage cell polarizations approaching those obtained for weak discharges in sealed cells (0.7 – 0.8). For the more realistic case of small polarization losses and optical pumping cell polarization slightly less than the sealed cell ideal, *P*_stc_ = 0.6 – 0.7 should be attainable. Currently, our capability to realize the full benefits of the PPR scheme is limited by loss of polarization in compression.

Even if PPR is not employed, reduced polarization loss would still allow operation at lower flow rates, thus decreasing the optical pumping cell pressure. The residence time in an optical pumping cell of the same volume is increased only slightly because the decrease in OPC pressure offsets the lower flow rate. As an example, let us assume that *f*_c_ = 0.95 could be obtained at *F* = 0.08 cm^3^/s, which leads to *Q* = 0.008 kPa·L/s for *p*_stc_ = 100 kPa, and thus *p*_opc_ = 0.18 kPa. Under these optical pumping conditions we have observed *P*_opc2_ = 0.64, hence a storage cell polarization of 0.61 could be obtained for the presumed value of 0.95 for *f*_c_. With some sacrifice in compactness, larger optical pumping cells should allow OPC polarization approaching 70 % in a single pass. For the model presented in Sec. 5.1, such low loss would require an order of magnitude reduction in the term *S*/(*α****κ***^1/2^), which indicates that the key parameters are the surface area of the compressor head, the relaxation properties of the compressor materials, and the compression ratio. For the second stage of the compressor, the displacement volume could be significantly reduced without substantially degrading the performance of the compressor. Hence it should be possible to decrease the surface area of the compressor head, although this would require a different compressor or new construction for the present one. As for the source of relaxation, our measurements to date do not clearly identify the next step. Indeed, the ineffectiveness of coating the Teflon diaphragm with aluminum suggests that a flow-rate dependent fraction of the gas will be depolarized almost regardless of the material for the diaphragm coating. The relaxation may be an inherent property of the compression method that would be difficult to surmount, but further investigation is required before this conclusion can be drawn. Finally, an increase in the compression ratio could be attempted, but a substantial increase is unlikely for this type of compressor. Realizing nearly lossless compression may require an alternative approach such as peristaltic compression [75], but detailed results for this method are needed.

Our results are also limited by the relatively high optical pumping cell pressure, especially for pure ^3^He. If the polarization loss could be reduced, a third stage of compression could substantially reduce the ultimate pressure. Reducing the elevation of pressure with flow requires increased pumping speed, which could be obtained by using two heads in parallel.

The performance of this apparatus is not significantly limited by laser power, but improving the results will require optimizing every aspect of the system. Although higher power than we have obtained has been reported for arc-lamp pumped Nd:LMA lasers [14], we have found that the attainable performance is variable and seems to be linked to the quality of each laser rod. Yb-doped fiber lasers [76,77] promise to provide higher power in a smaller, more compact system.

## Figures and Tables

**Fig. 10:**
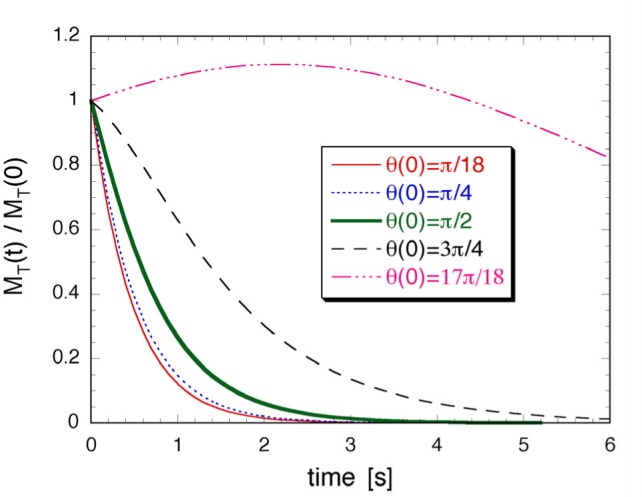
*M*_T_(*t*)/*M*(0) determined from Eq. (6) for tip angles of π/18, π/4, π/2, 3π/4, and 17π/18, where we have assumed *T*_2_ = 1 s and **τ**_∞_/*T*_2_ = 0.9. The nuclei are presumed to be in the lower energy state before the tip.

**Fig. 11:**
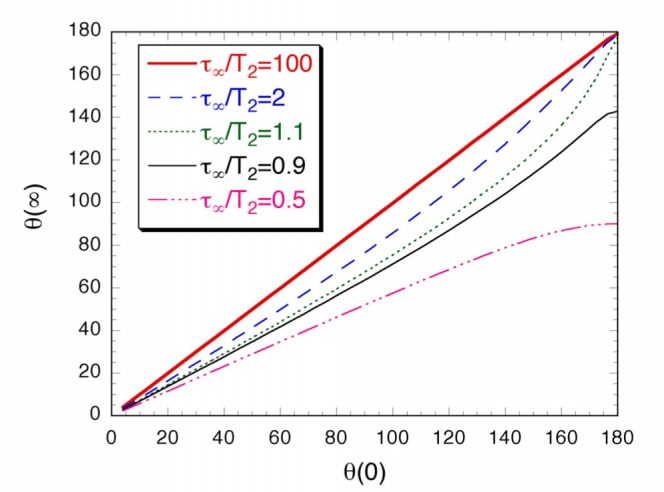
The variation of *θ* (∞) with *θ* (0), for **τ**_∞_/*T*_2_ equal to 100, 2, 1.1, 0.9, and 0.5.

## Footnotes

1 Certain commercial equipment, instruments, or materials are identified in this paper to foster understanding. Such identification does not imply recommendation or endorsement by the National Institute of Standards and Technology, nor does it imply that the materials or equipment identified are necessarily the best available for the purpose.

## 7. Appendix A. Radiation Damping in Free Induction Decay

For large magnetization and high quality factor pickup coils, radiation damping may affect free induction decay signals. Although these effects can be avoided, it is useful to be aware of the expected changes in the FID, which depend on the initial energy state of the nuclei, the strength of the damping, and the size of the initial tip angle. We begin by reproducing the solutions for free induction decay in the presence of radiation damping that are presented by Bloom [71]. In that work, the FID in the absence of radiation damping is assumed to be a simple exponential, and longitudinal relaxation is neglected. The initial tip of the magnetization is assumed to be instantaneous, ie. the effect of radiation damping during a finite rf pulse is not considered. For a initial magnetization *M*(0) and initial tip angle θ(0), the transverse magnetization *M*_T_(*t*) is given by

$$M_{T}(t)=M(0)\left(\frac{\tau_{\infty }}{\tau }\right)\text{sech}[(t-t_{m})/\tau ]$$ (6)

and the longitudinal magnetization for *t* =∞, *M*_z_(∞), is given by

$$M_{z}(\infty )=M(0)\tau_{\infty }\left(\frac{1}{\tau }-\frac{1}{T_{2}}\right)$$ (7)

where **τ**_∞_ is the radiation damping relaxation time for *T*_2_ =∞, *T*_2_ is the transverse relaxation time for **τ**_∞_=∞, is determined by

$${\left(\frac{\tau_{\infty }}{\tau }\right)}^{2}={\left(\frac{\tau_{\infty }}{T_{2}}\right)}^{2}+2\left(\frac{\tau_{\infty }}{T_{2}}\right)\cos\theta (0)+1$$ (8)

and *t*_m_ is determined by

$$\exp(2t_{m}/\tau )=\frac{1-[(\tau /T_{2})+(\tau /\tau_{\infty })\cos\theta (0)]}{1+[(\tau /T_{2})+(\tau /\tau_{\infty })\cos\theta (0)]}.$$ (9)

**τ**_∞_ is given by [2π*QfγM*(0)]^−1^, where *Q* is the quality factor of the pickup coil and *f* is the filling factor. As expected, **τ**_∞_ is smaller, hence radiation damping effects are larger, for large values of *Q*, *f*, or *M*(0). Figure 10 shows *M*_T_(*t*)/*M*(0) determined from Eq. (6) for five tip angles, where we have assumed *T*_2_ = 1 s and **τ**_∞_ = 0.9 s. The nuclei are presumed to be in the lower energy state before the tip. (A tip of (0) from the lower energy state is equivalent to a tip of π − *θ*(0) from a sample of nuclei initially in the higher energy state.) In the presence of radiation damping, the FID following a small tip from the lower energy state decays nearly exponentially with a shortened time constant. One can show that the decay is always exponential for small tips from the lower energy state, with a time constant given by $\tau^{-1}\approx \tau_{\infty }^{-1}+T_{2}^{-1}$. In contrast, the shape of the FID following a small tip from the higher energy state may be dramatically changed. Although *M*_T_(0)is always equal to *M*(0)sin *θ*(0), *M*_T_(*t*) may initially increase with time.

After the FID, the remaining magnetization will not be equal to *M*(0) cos *θ*(0), hence error may result if the tip angle is determined from its magnitude. We define an effective tip angle *θ*_∞_ = arccos[*M*_z_(∞)/*M*(0)]. Figure 11 shows the variation of *θ*(∞) with *θ*(0), for five values of **τ**_∞_/*T*_2_. For values of **τ**_∞_ > *T*_2_, *θ* (∞) for *θ* (0) π = is less influenced by radiation damping as compared to *θ* (∞) for ∞ (0) = π/2. For example, for **τ**_∞_/*T*_2_ = 1.1, *θ*(0) must be increased to 0.66π to yield *θ*(∞) = π/2, whereas *θ*(∞) = π will be observed for a tip angle only slightly greater than *θ*(∞) = π. Hence in the presence of relatively weak radiation damping, it should be more accurate to determine the rf power required for a π/2 pulse by using half the value of rf power that is found to produce a π pulse, rather than determining the rf power required for complete destruction of the magnetization. Alternatively, the optical signal from low pressure gas will not suffer from radiation damping issues because of the small magnetization.

We determined cell relaxation times by measuring the FID signal vs. time using small tip angles and applying a small correction for the magnetization destroyed in each tip. In the presence of radiation damping, this correction should use *θ*(∞) rather than *θ*(0). Fig. 11 clearly shows that the effective tip angle is less affected when the nuclei are initially in the lower energy state.
